# Clinical features of headache patients with fibromyalgia comorbidity

**DOI:** 10.1007/s10194-011-0377-6

**Published:** 2011-08-17

**Authors:** Marina de Tommaso, Antonio Federici, Claudia Serpino, Eleonora Vecchio, Giovanni Franco, Michele Sardaro, Marianna Delussi, Paolo Livrea

**Affiliations:** 1 Neurophysiopathology of Pain Unit, Neurological and Psychiatric Sciences Department, Medical Faculty, Policlinico General Hospital, Aldo Moro University, Neurological Building, Piazza Giulio Cesare 11, 70124 Bari, Italy; 2 Physiology and Pharmacology Department, Aldo Moro University, Bari, Italy

**Keywords:** Primary headache, Fibromyalgia, Comorbidity

## Abstract

Our previous study assessed the prevalence of fibromyalgia (FM) syndrome in migraine and tension-type headache. We aimed to update our previous results, considering a larger cohort of primary headache patients who came for the first time at our tertiary headache ambulatory. A consecutive sample of 1,123 patients was screened. Frequency of FM in the main groups and types of primary headaches; discriminating factor for FM comorbidity derived from headache frequency and duration, age, anxiety, depression, headache disability, allodynia, pericranial tenderness, fatigue, quality of life and sleep, and probability of FM membership in groups; and types of primary headaches were assessed. FM was present in 174 among a total of 889 included patients. It prevailed in the tension-type headache main group (35%, *p* < 0.0001) and chronic tension-type headache subtype (44.3%, *p* < 0.0001). Headache frequency, anxiety, pericranial tenderness, poor sleep quality, and physical disability were the best discriminating variables for FM comorbidity, with 81.2% sensitivity. Patients presenting with chronic migraine and chronic tension-type headache had a higher probability of sharing the FM profile (Bonferroni test, *p* < 0.01). A phenotypic profile where headache frequency concurs with anxiety, sleep disturbance, and pericranial tenderness should be individuated to detect the development of diffuse pain in headache patients.

## Introduction

According to the American College of Rheumatology (ACR), fibromyalgia (FM) is a chronic pain syndrome of unknown aetiology, characterized by diffuse pain for more than 3 months and tenderness in at least 11 tender point sites out of 18 [1]. Despite these apparently simple diagnostic criteria, the syndrome appears more complex with associated symptoms including non-restorative sleep, fatigue, and cognitive dysfunction [2].

Causes of FM are largely unknown although there is a growing body of evidence to support central sensitization mechanism underlying chronic musculoskeletal pain in these patients [3]. Although the association between FM and primary headaches is almost frequent, rheumatologists have classified it as “an unexplained clinical condition” [4].

FM comorbidity was specially studied in migraine population, with a prevalence of 35.6% in patients with transformed migraine [5] and 22% in episodic migraine patients [6]. In our cohort of 217 consecutive headache patients, 36.4% of the patients were found to have FM [7]. FM was the most common among chronic migraine and chronic tension-type headache patients. Headache frequency, pericranial muscle tenderness, anxiety, and sleep inadequacy were especially associated with FM comorbidity. Tension-type headache was the most common primary headache associated with FM, with a 59.01% prevalence, compared with episodic and chronic migraine, presenting with 28.8% prevalence. There was no difference between chronic tension-type headache and chronic migraine in FM syndrome prevalence; this suggests that FM is a syndrome complicating these two types of chronic headaches. On the other hand, headache is common among the patients with FM. In a study of 100 patients with FM, recurring headache occurred in 76%, and predated the onset of FM, on average, 7 years before the onset of FM symptoms [8]. Similarly, in a study of 33 FM patients, current migraine was present in 45% and a lifetime history of migraine in 55% [9].

The mutual comorbidity between headache and FM reserves much attention, in view of common pathophysiological basis [10] and problems connected with therapeutical approach [11].

In addition, there are still unresolved questions, e.g. the prevalence of FM in other primary-headache forms, as trigeminal autonomic cephalalgias (TACs), and the factors favouring FM comorbidity. We aimed to extend our study [7] to a larger sample selected during a total observational period of 2 years at our tertiary headache centre, to check the validity of previously observed prevalence of FM comorbidity and to characterize the features of patients sharing headache and FM syndrome and their representation within main headache groups and types, in an attempt to give further details on FM comorbidity in less common forms of primary headaches.

## Methods

Following previous evaluation from 1 January 2007 to 30 June 2007, where a total of 274 patients were screened and 217 were included [7], we screened further 849 consecutive outpatients, who came for the first time at the Neurophysiopathology of Pain Unit (Neurological and Psychiatric Sciences Department, Bari University) from 1 July 2007 to 30 December 2009. The Neurophysiopathology of Pain Unit is a tertiary referral centre where patients are referred by primary physicians as well as by neurological and other specialty clinics. All participants gave written informed consent after receiving a detailed explanation of the purpose and design of the study. The study was approved by the local Ethics Committee of the Policlinico General Hospital.

According to the previous study [7], during the first visit, all subjects had a standardized interview and underwent clinical neurological and psychiatric examination. The inclusion criteria was a diagnosis of primary headache made by three neurologists with special experience in headache, according to the International Classification of Headache Disorders, 2nd edn (ICHD-II) criteria [12], and was supported by a 3-month observation time with a headache diary and allodynia questionnaire.

The inclusion/exclusion criteria and clinical management of patients were the same as the previous study [7]. Briefly, patients with general medical, neurological or psychiatric diseases [13], were excluded from the study, as well as the patients on central nervous system-active drug therapy to rule out any drug effect on diffuse pain. A particular attention was taken in screening out patients suffering from various conditions with diffuse pain, such as arthritis, diabetes or other metabolic causes of neuropathic pain. We included other types of primary headaches, and in the case of hemicrania continua (code. 4.7) [12], the 3 months preceding the first visit were considered for headache features and FM comorbidity, to prescribe indomethacine and confirm the diagnosis in the next control.

During the follow-up visit (except for patients with hemicrania continua, who were examined during their first visit, and the diagnosis confirmed at the follow-up), all patients underwent the clinical assessment, defined in the previous study [7], consisting of evaluation for FM diagnosis and tender point count [1], frequency of headache [7], total tenderness score (TTS) [14], allodynia questionnaire [15, 16], Short-Form 36 (SF-36) Health Survey [17], depression [self-rating depression scale (SDS)] and anxiety [self-rating anxiety scale (SAS)] scales [18, 19], Multidimensional Assessment of Fatigue (MAF) [20], and Medical Outcomes Study (MOS) [21]. In this study, we considered the sleep problems index (SLP9), expressing the sleep problems index, and Sleep quantity (SLPQ), expressing the sleep quantity [21].

Migraine Disability Assessment scale (MIDAS) [22], in the Italian version [23], was used to quantify headache-related disability in all headache patients, differently from the preliminary study [7]; the MIDAS score was considered only for migraine groups.

Patients presenting with FM comorbidity, according to the ACR criteria [1], were submitted for the Manual Tender Point Survey, [24–26] and answered the FM Impact Questionnaire (FIQ) [27], in accord with the previous study [7].

## Statistical analysis

All patients were included in headache major groups, according to the main ICHD-II codex [12], where we did not include the mixed forms. Within each major group, the type of headache was further specified, and subgroups including at least ten patients were considered. The frequency of FM across main headache groups and types was checked by means of the Pearson’s Chi-square as well as the distribution of FM comorbidity between genders. The clinical variables such as age, headache frequency and duration, allodynia, SAS, SDS, MAF, MIDAS, SLP9, MOS, and TTS, were introduced in a multivariate analysis (MANOVA) with type III sum of square where the comorbidity for FM was the main factor. The least significant difference (LSD) was applied to the confidence intervals of the single variables. To find the best separating variables between FM and non-FM groups, a stepwise discriminant analysis was run, using Mahalanobis distance, *F* probability of 0.05 for entry and 0.1 for removal, classification function coefficient by means of Fisher’s linear discriminant test, and leaving one out of final classification. To further specify if a specific group or type of headache shared the clinical profile of FM, the function coefficient was then employed to attribute to each patient the probability of membership to the FM group. The Bonferroni test for multiple comparisons was used to detect the main differences of FM probabilities across headache groups and types. We further evaluated if there was a correlation between the gravity of headache, expressed by MIDAS, frequency, allodynia, and TTS; and the severity of FM symptoms, expressed by the FIQ and total tender point survey score, by means of the Pearson’s correlation test. All statistics were done applying the SPSS version 8.

## Results

Among a total of 1,123 patients who came for the first time to our centre, we included 889 consecutive patients (204 men). The remainder 224 were not included for various reasons; 24 [3 cluster headache, 1 migraine with aura (MA), 20 chronic migraine] needed to start or modify the preventive treatment as soon as possible; a very invalidating headache or various familiar or social circumstances did not enable the 3 months observation. Two patients were pregnant, 50 patients were affected by secondary headaches, 100 did not pass the inclusion criteria for various reasons, as psychiatric or general medical comorbidities or CNS acting drugs intake, the remainder were lost to follow-up or did not apply to diary compilation.

All patients were included in four headache major groups, according to the main ICHD-II codex [12] (Table 1). To understand if FM comorbidity involved preferentially a form of primary headache, in this subdivision we did not consider mixed forms across different headache major groups. Within each major group, the type of headache was further specified, and subgroups including at least ten patients were considered. We obtained ten headache types subgroups, with an eleventh mixed-type group (Table 2).

**Table 1:** Frequency of fibromyalgia (FM) comorbidity in the primary headache groups

	No FM (no.)	FM (no.)
Main ICHD II group		
Cod 1.00 migraine	521	113
Cod 2.00 tension-type headache	100	54
Cod. 3.00 cluster headache and other TACs	24	
Cod.4.00 other primary headaches	35	2
Total	680 (80.1%)	169 (19.9%)

Pearson chi square: 34.77, *df* 3, *p* 0.0001

**Table 2:** Frequency of fibromyalgia comorbidity (FM) in the primary headaches types

Primary headache type	No FM (no.)	FM (no.)
Chronic migraine cod.1.5.1	88	53
Chronic tension-type headache cod 2.3	54	43
Cluster headache cod 3.1	13	
Episodic frequent tension type headache cod 2.2	46	9
Hemicrania continua cod 4.7	12	1
Migraine with aura cod 1.2	20	
Migraine with aura plus migraine without aura cod 1.1 plus 1.2	35	6
Migraine without aura cod 1.1	377	55
Migraine without aura plus frequent episodic tension type headache cod 1.1 plus 2.2	36	6
Mixed headache types		
Primary stabbing headache no. 7 cod 4.1	23	1
Primary thunderclap headache no. 1 cod 4.6		
Hypnic headache no. 8 cod 4.5		
Primary cough headache no. 4 cod 4.2		
Primary exertional headache no. 3 cod 4.3		
Paroxysmal hemicrania cod 3.2	11	
Total	715 (80.43%)	174 (19.57%)

Pearson chi square: 96.92, *df* 10, *p* 0.0001

In Table 3, the main clinical features of each headache type are reported. Considering the main headache groups, FM prevailed in tension-type headache, followed by migraine group (Table 1). Considering the primary headache types, FM was specially represented in chronic tension-type headache, followed by chronic migraine (Table 2). Among FM patients, 13 were men (7.4% of all the FM patients, vs. 22.94%) in the entire headache population (Pearson’s Chi-square 29.38; *df* 1, *p* 0.0001). The whole considered variables significantly distinguished headache patients from those without FM comorbidity (results of MANOVA: *F* = 21.41, error *df* 875; *df* 13, *p* 0.0001). Allodynia symptoms and total hours of sleep were not significantly different between patients presenting and not presenting with FM comorbidity (Table 4).

**Table 3:** Clinical characteristics of the primary headache types

Primary headache type	Age (years) M (SD)	Duration (years) M (SD)	Frequency (days/headache/month) M (SD)	Sex (no.)
Chronic migraine cod.1.5.1	41.82 (13.36)	17.67 (13.61)	24.16 (6.45)	M 19 F 122
Chronic tension-type headache cod 2.3	45.81 (15.60)	11 (12.4)	23.13 (6.51)	M 31 F 66
Cluster headache cod 3.1	41 (9.89)	17.8 (14.71)	13.8 (8.13)	M 9 F 4
Episodic frequent tension type headache cod 2.2	41.6 (15.6)	11.6 (13)	5.5 (3.2)	M 14 F 41
Hemicrania continua cod 4.7	49.61 (15.86)	11.3 (11)	28.5 (1.21)	M 2 F 11
Migraine with aura cod 1.2	36.4 (11.26)	13.7 (9)	1.8 (1.12)	M 6 F 14
Migraine with aura plus migraine without aura cod 1.1 plus 1.2	35.56 (11.71)	18.25 (13.20)	8.4 (7.5)	M 7 F 34
Migraine without aura cod 1.1	37.26 (12.56)	13.7 (9)	5.41 (3.2)	M 96 F 336
Migraine without aura plus frequent episodic tension type headache cod 1.1 plus 2.2	39.71 (13.38)	17.8 (11.9)	8.4 (7.2)	M 8 F 34
Mixed headache types				
Primary stabbing headache no. 7 cod 4.1	37 (14.98)	7.7 (9)	8.4 (8.91)	M 7 F 17
Primary thunderclap headache no. 1cod 4.6				
Hypnic headache no. 8 cod 4.5				
Primary cough headache no. 4 cod 4.2				
Primary exertional headache no. 3 cod 4.3				
Paroxysmal hemicrania cod 3.2	43.5 (13.2)	9.9 (9.8)	19.4 (11.3)	M 5 F 6

Means and standard deviations of clinical variables in primary headaches types

**Table 4:** Clinical features of fibromyalgic patients

Dependent variable	Mean	Lower bound	Upper bound
Age			
No FM	37.291	36.192	38.390
FM	45.662	43.429	47.894
Duration			
No FM	14.934	13.866	16.001
FM	18.035	15.866	20.203
Frequency			
No FM	19.354	17.769	20.938
FM	28.428	25.211	29.645
MIDAS			
No FM	33.882	30.032	37.733
FM	51.992	44.174	59.810
Allodynia			
No FM	3.390	3.057	3.723
FM	4.108	3.432	4.783
TTS			
No FM	4.187	3.710	4.663
FM	9.992	9.025	10.960
MAF			
No FM	49.433	46.458	52.408
FM	77.500	71.459	83.541
SAS			
No FM	40.170	39.459	40.880
FM	48.462	47.019	49.904
SDS			
No FM	38.780	38.020	39.540
FM	45.754	44.210	47.298
ISF			
No FM	42.623	41.856	43.390
FM	37.085	35.528	38.641
ISM			
No FM	42.153	41.200	43.106
FM	36.469	34.533	38.405
SLP9			
No FM	33.116	30.708	35.524
FM	53.631	47.715	59.547
SLPQ			
No FM	6.503	6.328	6.677
FM	6.107	5.678	6.536

Clinical variables introduced in the multivariate analysis to compare headache patients with and without fibromyalgia (FM) comorbidity. The least significant difference (LSD) was beyond the 0.05 level for all variables except for allodynia and SLPQ items

**Fig. 1:**
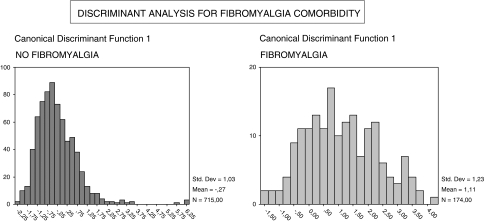
The figure summarizes the classification of non-fibromyalgic and fibromyalgic headache patients, according to the discriminating factor derived from the best separating variables (frequency of headache, self-rating anxiety scale, total tenderness score, sleep problems index, physical component summary)

The stepwise discriminant analysis found that the best discriminating variables for FM comorbidity were frequency of headache, anxiety levels, TTS, sleep disturbances, and physical component of life quality (Table 5). The canonical discriminant function, correctly classified 81.2% of the original grouped cases and 80.5% of the cross-validated grouped cases (Fig. 1).

**Table 5:** Classification function coefficients

	No FM	FM
Frequency	0.082	0.102
SAS	0.865	0.928
TTS	0.052	0.301
SLP9	−0.03	−0.002
PCF	0.891	0.831
Constant	−37.382	−41.132

Fisher’s linear discriminant functions

Discriminating variables between fibromyalgic (FM) and not fibromyalgic patients

*SAS* self-rating-anxiety-scale, *TTS* total tenderness score, *SLP9* sleep problems index, *PCF* physical component summary

**Fig. 2:**
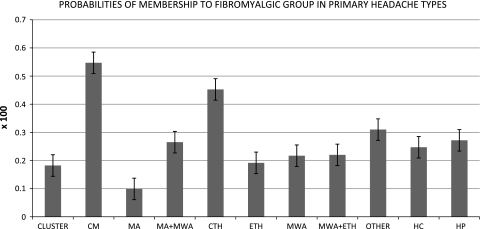
Probabilities (mean ± standard error) of membership to FM groups for patients included in headache types (*CM* chronic migraine, *MA* migraine with aura, *MWA* migraine without aura, *CTH* chronic tension-type headache, *ETH* episodic tension-type headache, *HC* hemicrania continua, *HP* paroxysmal hemicrania), according to the discriminating function. The results of Bonferroni test, revealed that CM and CTH groups significantly differed from the others (*p* < 0.01), for the highest probability to share the FM profile, while MA differed from the others for the lowest probability (*p* < 0.05)

The probability of membership to FM group did not differ significantly across the main headache groups, while both chronic migraine and chronic tension-type headache patients exhibited the highest and MA patients the lowest levels of probability (Fig. 2).

The FIQ was positively correlated with frequency of headache, MIDAS score, and allodynia, while pain at the tender points was correlated with TTS and frequency of headache (Table 6).

**Table 6:** Correlation between fibromyalgia and headache indices of severity in the 174 patients presenting with fibromyalgia comorbidity

	Frequency	MIDAS	Allodynia	TTS
FIQ				
Pearson correlation	0.289	0.215	0.349	0.065
Sig. (two-tailed)	0.001	0.014	0.000	0.467
Tender point survey				
Pearson correlation	0.186	0.036	0.163	0.405
Sig. (two-tailed)	0.03	0.683	0.052	0.000

*FIQ* Fibromyalgia Impact Questionnaire *TTS* total tenderness score

## Discussion

### Frequency of FM comorbidity in headache groups and types

In this study, which is the extension of the previous one in a smaller headache series [7], we found a lower frequency of FM representation in the selected patients. Other studies on this topic were specially dedicated to chronic or episodic migraine without aura (MWA), with a reported frequency, respectively, of 35 and 22% in the selected populations [5, 6]. A recent multicentre study on 1,413 patients [28], reported 10% of migraine patients presenting with FM comorbidity, but the features of migraine were not specified. The frequency of 17.8% that we actually found in the migraine group was almost the same as the previous report [7], with a minimum in purely MA and a maximum in chronic migraine. The apparent discordance of FM prevalence across studies may be due to variability in applying FM diagnostic criteria, or the uncertainness of a story of widespread pain reported from patients who came to visit for another reason. The FM diagnostic criteria are not devoid of problems, and the ACR has proposed to enlarge the symptoms useful for diagnosis [29], applying clinical criteria based on fatigue, cognitive symptoms, and the extent of somatic symptoms, without considering the number of positive tender points. These new criteria would be easily applied in headache centre and facilitate the detection of FM comorbidity. Interestingly, FM comorbidity was absent in patients presenting exclusively with MA attacks. This is a new data, given that in the study by Ifergane et al. [6] and Tietjen et al. [28], the presence of aura was reported without specifying the contemporary occurrence of MWA attacks. In the present study, patients with both MA and MWA diagnosis had the same FM frequency as those without aura, while it seemed that it was the exclusive presence of MA attacks to preserve from FM comorbidity. These data need to be confirmed in larger series, and may be supported by a pathophysiological explanation, as also supposed below. No studies are available on FM prevalence in the other forms of primary headache. Even in tension-type headache, where growing evidences indicate common pathophysiological basis with FM, only single cases of comorbidity are reported [30–34]. In our study, according to the previous one [7], tension-type headache showed the major FM representation among primary headaches, with 35.1% prevalence. In FM populations, both migraine and tension-type headache are considered among the main causes of comorbidity [4]. The present results indicate a 25% frequency of FM in migraine and tension-type headache groups, not largely dissimilar from our preliminary study [7], in accord with which the chronic forms share the highest FM representation. This may also partly explain the preponderance of patients associating tension-type headache and generalized pain, given that in the tension-type group, most of the patients were chronic. It was the low representation of FM patients in the other primary headache groups (TACs and other forms) that reduced the FM frequency found in the total headache sample. For hemicrania continua, the retrospective evaluation used to prescribe indomethacine for confirming diagnosis after the observational period, may have induced an underestimation of FM comorbidity, with respect to the other considered headache types. Also, taking into consideration the low number of patients included in groups three and four of primary headaches, no definitive conclusion about FM comorbidity could be made, rather an impression of a low FM representation even in types with high headache frequency was found. In this sense, the frequency of headache should be the main but not the exclusive factor favouring FM, as specified below.

### Factors favouring FM comorbidity

The phenotype expression of headache patients complaining with FM comorbidity included higher headache frequency, anxiety, pericranial tenderness, reduced physical performances, and sleep disturbances. Allodynia, which expresses the severity of central sensitization occurring during headache episodes [38], was not significantly increased in our FM series, suggesting that central sensitization should persist outside acute headache and generate myofascial pain to favour FM comorbidity. As expected, women prevailed in the FM group, as FM is six times more common in women, while headache and specially migraine is three times more common. The cycle phase would also influence pericranial and somatic tender points sensitivity [39], though in the present study this aspect was not taken into consideration. This is a confirmation of discriminating features of FM previously detected in a smaller headache series [7] with the inclusion of physical component of quality of life. Chronic migraine and chronic tension-type headache subtypes shared this headache profile in a significant way with respect to the other forms, confirming headache frequency as the primary factor for FM comorbidity. Pericranial tenderness is considered as a consequence of chronic headache [36, 37], as a sign of permanent sensitization at cervical and trigeminal second-order nociceptive neurons, subtended by a pathogenic process similar to that causing pain at tender points [32]. Reduced habituation to pain, common to migraine and FM [10], may facilitate central sensitization and myofascial pain persistence in the presence of other favouring conditions such as anxiety and sleep disturbances. A self-outstanding circuit of increased headache frequency, development of pericranial myofascial pain, persisting central sensitization with somatic diffusion of pain, may explain FM comorbidity in both chronic tension-type headache and chronic migraine, where the persistence of pericranial tenderness contributes to the transformation from episodic into chronic form [40]. Sleep disturbance is a well-recognized factor in FM syndrome [35], and our results confirm that in headache patients it favours generalized myofascial pain. The total numbers of sleep hours were not dissimilar between FM and non-FM patients, while the quality of sleep was the discriminating factor for FM in our headache series, in accord with our previous reports [7]. Clinical and preclinical data concur that sleep disruption causes hyperalgesia, and despite widely distributed and overlapping neural networks, regulate states of sleep and pain; and the brain mechanisms through which sleep and pain interact, remain poorly understood [41, 42]. There is an intriguing hypothesis that sleep deprivation decreases the analgesic effect of distraction in healthy individuals [43], and in the case of migraine, it may accentuate the pattern of altered pain modulation under distracting factors [44]. There are also evidences that rapid eye movement (REM) sleep deprivation is especially linked to hyperalgesia [45]. A significant association between severe sleep disturbances and chronic headache [46, 47] and central sensitization [48] has further been reported. Poor quality of sleep promotes diffusion of myofascial pain in headache patients, but which sleep phase is more involved in the generation of widespread pain remains to be clarified. Despite FM patients exhibiting higher depression and anxiety levels, it was the latter feature that best discriminated patients with diffuse pain among our headache population. Mongini et al. [49] found that the presence of anxiety considerably increases the level of muscle tenderness in the head and, even more, in the neck, and might facilitate the evolution into chronic headache forms. In this way, anxiety may also facilitate diffuse myofascial pain and FM comorbidity in headache patients presenting with higher pericranial muscle tenderness. FM patients were also characterized by a reduced functioning in daily living, inherent to physical abilities. This may suggest that persisting pericranial and somatic myofascial pain have a consequence on motor performances and that physical inability mainly compromise quality of life in patients sharing FM comorbidity. A combination of symptoms is needed to favour FM comorbidity, headache frequency being the main, though not the only cause. In fact, other primary headache types such as cluster headache, hemicrania continua, or parossistic migraine presented with high headache frequency and low probability do match the clinical profile of FM patients. However, the low number of patients included in these types of primary headaches deserves further confirmation in a larger series. Purely MA patients presented with the lowest probability to share the FM profile, while the combination with migraine attacks not preceded by aura symptoms conditioned higher representation of features facilitating diffuse somatic pain. Tietjen et al. [28] recently found that the presence of aura did not preserve the patients from FM comorbidity who were presenting with both types of migraine. Acute central sensitization phenomena were firstly described as a development of migraine aura [50] and allodynia has been confirmed a usual symptom in this type of migraine [28]. Moreover, acutely occurring allodynia does not account for FM comorbidity, which is present when central sensitization persists outside attacks and determines pericranial tenderness. This argument needs, in our opinion, further evaluation to specify if the prevalent presence of aura characterizes a migraine phenotype with low predisposition to chronic pain.

According to the previous report [7], headache severity concurs with FM gravity, as expressed by the positive correlation between the MIDAS and the impact of FM on life functions. Although the findings reported by Marcus et al. [8] did not support headache as an aggravating factor for FM, our data confirm that when headache is present its severity is linked to an increase in expression of FM symptoms. Our cases probably represent a subpopulation among FM patients, reporting headache as the most relevant problem, though in our opinion the relevance of headache features deserves much attention in FM series, for the large frequency of this symptom [35]. Allodynia expressed during acute headache, correlated with the invalidity linked with diffuse pain. This correlation may suggest that the central sensitization phenomena occurring during headache may also worsen the sufferance linked with fibromyalgic pain. An increased activation of the nociceptive system at central level may be a generalized phenomenon explaining a more severe impairment derived from diffuse muscle-skeletal pain. In future studies, it would be interesting to evaluate if the transformation of headache into whole-body allodynia/hyperalgesia during a migraine attack, mediated by sensitization of thalamic neurons, may be an aggravating factor for FM [51].The degree of evoked pain at tender points, was correlated with pericranial tenderness, confirming that both symptoms are subtended by analogous mechanisms of muscular hyperalgesia [32]. Frequency of headache seemed to concur with augmented pain evoked at tender points, suggesting that a generalized increment of pain sensitivity may develop with the increase in headache occurrence [36]. A more robust correlation should be confirmed in larger multi-centre studies.

## Conclusions

The overall consideration derived from the present data, is that the evaluation of FM comorbidity may increase the knowledge about the basic mechanisms of chronicization and the expression of central sensitization phenomena in the different primary headache subtypes. Though we have to acknowledge the weakness of the study, being conducted in a single tertiary referral centre, not representing the general population, the detection of a phenotypic profile, where headache frequency concurs with anxiety, sleep disturbance, and pericranial tenderness would be useful in the management of diffuse pain and physical invalidity development.
